# Optimization of Resolving Power, Fragmentation, and Mass Calibration in an Orbitrap Spectrometer for Analysis of 24 Pesticide Metabolites in Urine

**DOI:** 10.1155/2019/1917369

**Published:** 2019-04-17

**Authors:** Pablo Dualde, Clara Coscollà, Agustin Pastor, Vicent Yusà

**Affiliations:** ^1^Foundation for the Promotion of Health and Biomedical Research in the Valencian Region, FISABIO-Public Health, 21 Avenida Catalunya, 46020 Valencia, Spain; ^2^Analytical Chemistry Department, University of Valencia, Edifici Jeroni Muñoz, Dr. Moliner 50, 46100 Burjassot, Spain; ^3^Public Health Laboratory of Valencia, 21 Avenida Catalunya, 46020 Valencia, Spain

## Abstract

Mass spectrometer parameters such as Resolving Power, type of fragmentation, and mass calibration mode were optimized in the analysis of 24 pesticide metabolites in human urine using Ultra-High Pressure Liquid Chromatography coupled to Orbitrap High-Resolution Mass Spectrometer (UHPLC-HRMS). The best results were achieved with a Resolving Power of 25,000 FWHM and by applying Collision Induced Dissociation fragmentation mode (40 eV).

## 1. Introduction

The ever growing number of chemicals being used, such as pesticides, care products, UV filters, parabens, and so on, has an impact on the environment and therefore on humans, especially in vulnerable populations [1]. To protect consumers from such contaminants, an evaluation of the exposure to these chemicals and the subsequent risk assessment is necessary. The National Report on Human Exposure to Environmental Chemicals in the United States [2] and the Human Biomonitoring Report of Environmental Chemicals in Canada [3] are both examples of biomonitoring programs that assess the exposition of a population to environmental chemicals over time. The European Environment and Health Strategy also encourages the adoption of human biomonitoring studies across Europe [4].

Some pesticide metabolites are biomarkers of pesticide exposure. These metabolites are present in urine at concentrations of few ng mL^−1^ [2], and consequently metabolite determination requires sensitive and selective analytical methods. The usual analytical technique for polar metabolites in human biomonitoring studies is the LC-MS/MS [5]. However, the introduction of the high-resolution (>10,000 FWHM) mass spectrometers, which allow to obtain mass accuracies lower than 5 ppm, such as Q-TOF or Orbitrap, has allowed the implementation of combined quantitative target and postrun target analytical strategies for comprehensive determination of pesticides and other emerging contaminant metabolites [6].

Over the last few years, liquid chromatography coupled to Orbitrap high-resolution mass spectrometry (LC-HRMS) has been applied in human biomonitoring studies [6]. Recently, Cortejade* et al*. (2016) developed an analytical method for the targeted screening and multiresidue quantification of 38 contaminant metabolites in urine, including 12 pesticides, a pesticide metabolite (tributyl phosphate), and other compounds of different families [7]. Likewise, Roca* et al*. (2014) developed an analytical method that combined the quantitative target analysis of urinary metabolites of pesticides with a retrospective analysis using liquid chromatography coupled to HRMS. In this study, the main factors governing the ion-source ionization were optimized [6]. In addition,* López et al.* (2016) developed a retrospective analytical methodology for the analysis of pesticide metabolites in urine by LC-HRMS [8].

In the existing literature on application of HRMS to food and feed contaminants [9–14] and in the field of human biomonitoring [6, 15–21], the emphasis so far has mainly been the detectability of the analytes based on retention time and the exact mass of the most abundant analyte ion and on quantitative determination. However, Resolving Power (R) and mass calibration should be further investigated in order to avoid interferences and improve mass accuracy. Likewise, the selection of the fragmentation mode (High-energy Collision Dissociation, HCD, or Collision Induced Dissociation, CID) and their optimization could improve the sensitivity of the confirming ions (fragments).

How much Resolving Power is necessary to apply to a specific problem should be a frequent analytical question [22]. Orbitrap mass spectrometers allow a wide range of Resolving Powers. Theoretically, a higher Resolving Power provides a better resolution of analytes from isobaric interferences present in complex matrices such as urine. However, in Orbitrap, greater Resolving Power requires longer measurement time. A higher Resolving Power leads to the monitoring of fewer data points per time unit [23]. Therefore, for quantitative analysis, the Resolving Power for each matrix-analyte combination should be selected taking into account the isobaric interferences and the number of data points required for provide good peak shape. The Resolving Power has been studied in the literature for veterinary drugs in food and animal samples [22] as well as for other residues and contaminants in food and feed [24].

In order to obtain a suitable mass accuracy (< 5 ppm) in an Orbitrap spectrometer, a proper mass calibration must be employed. External mass calibration is performed previously to the analysis by direct infusion of a mix of compounds with known masses; the experimental m/z values obtained are then corrected with the theoretical m/z values in order to fit the accuracy of the analyzer. Until now, external mass calibration in Orbitrap has been widely employed in biological samples, food, and feed [6, 7, 11, 12, 18, 20]. However, as Leendet* et al.* (2015) have pointed out “improper external mass calibration can lead to large systematic errors in mass measurements” and “external mass calibration range must include the mass range of interest” [25]. Another strategy is the use of internal mass calibration, which is achieved by introducing a compound with a known theoretical m/z value (lock mass) during the analysis. The comparison between the theoretical and experimental errors is used to normalize the m/z values of the rest of peaks [26]. Thus, Strano-Rossi* et al*. (2015) successfully determined stimulants and drugs in food supplements using internal mass calibration in an Orbitrap system [27]. Internal calibration is recommended to avoid drifts of the external calibration over time [25].

Unlike conventional quadrupole (QqQ) instruments, Orbitrap Exactive™ users do not implement a compound-specific fragmentation optimization. In Exactive™, two all ion fragmentation (AIF) modes are allowed: HCD and CID [28]. In HCD, the ions are fragmented in a collision cell using N_2_ as a collision gas, while CID allows the ions dissociation through interaction with neutral target species. Consequently, some authors have studied the fragmentation, optimizing the HCD cell for different substances in biological samples, food and feed [6, 11, 12, 18, 20] in an Orbitrap detector. Optimization of HRMS (Orbitrap) fragmentation mode has also been employed in areas such as proteomics [28] or for the identification of oligosaccharides [29]. Optimization of the different types of fragmentation modes (HCD and CID) will probably improve the sensitivity of these methods.

In a previous work [6], we studied the influence of the HCD collision energy on the fragmentation of various pesticide metabolites in urine, working at 50,000 FWHM. In the present paper, we want to increase the speed of the analysis using the polarity switching function (ESI^+^ and ESI^−^ in the same injection) and to study more in depth other factors that can have a decisive influence on the sensitivity, accuracy and speed of the analysis of pesticide metabolites in urine. Consequently, the aims of the present work are to (i) study the influence of Resolving Power (R) on the signal intensity and mass accuracy of the different ions; (ii) compare the two all ions fragmentations (AIF) modes (HCD; CID); (iii) evaluate the two options for mass calibration (internal and external) to improve mass accuracy for this specific application.

## 2. Materials and Methods

This study has been developed in the framework of the DENAMIC project, which included all the required ethical approvals.

### 2.1. Reagents and Chemicals

Solvents were specific for pesticide residue analysis and of analytical grade. Acetonitrile and methanol were supplied by Scharlab (Barcelona, Spain). Acetic acid (purity 98-100%), *β*-glucuronidase arylsulfatase enzyme, and anhydrous sodium acetate were obtained from Merck (KGaA, Darmstadt, Germany). Deionized water was organically and biologically purified by using a Milli-Q Ultrapure System (Millipore, Darmstadt, Germany). QuEChERS EN extraction kits, containing 4 g MgSO_4_; 1 g NaCl; 1 g Sodium Citrate; 0.5g Sodium Hydrogencitrate Sesquihydrate, were obtained from Agilent Technologies (Madrid, Spain).

Standards of pesticide metabolites (Table 1) were achieved. All commercial standards were of high purity and were obtained from Dr. Ehrenstorfer (Augsburg, Germany), Sigma-Aldrich (Barcelona, Spain), Cerilliant-Certificated Reference Materials (Texas, USA), Cambridge Isotope Laboratories (Massachusetts, USA), Santa Cruz Biotechnology (Heidelberg, Germany), and AccuStandard (New Haven, USA). Stock standard solutions containing 20–500 mg L^−1^ of the individual compounds were prepared by weighing each compound and dissolving it in acetonitrile. Solutions were stored at −20°C. Multianalyte intermediate standard solutions were prepared by diluting the individual stock solutions in acetonitrile and used for preparing working mixed-standard solutions in acetonitrile: water (10:90, v/v). The concentration of the analytes in working solutions ranged from 1000 to 5000 ng·mL^−1^ depending on the compound.

### 2.2. Sample Preparation and UHPLC-HRMS

A previously developed sample preparation was used [6]. Briefly, in order to hydrolyze the possible glucuronide- or sulfate-conjugated metabolites, 5 mL of urine was mixed with 1 mL of 0.2 M acetate buffer 20 *μ*L of *β*-glucuronidase aryl sulfatase enzyme and the internal standard solution mix in the analysis of real samples. The samples were maintained at 37°C overnight for hydrolysis.

Metabolites were extracted from the urine samples employing the dispersive solid phase extraction QuEChERS kits. In a 50 mL polypropylene tube, the urine was mixed with 10 mL of acetonitrile, a QuEChERS pouch, and 2 ceramic pieces. After centrifugation, the acetonitrile phase was transferred and evaporated to dryness in a water bath at 37°C under a nitrogen stream. Subsequently, 200 *μ*L of methanol: water (10:90, v/v) containing 0.1% of acetic acid was added and the solution was transferred into a Millipore 0.2 *μ*m Eppendorf and ultra-centrifuged. The final extract was transferred into an injection vial and analyzed in the UHPLC-HRMS system.

Chromatographic separation was performed on an ultra-high performance liquid chromatography (UHPLC) system Accela™ equipped with a Hypersil Gold column (100 mm x 2.1 mm, 1.9 *μ*) from ThermoFisher Scientific (Bremen, Germany). The chromatographic separation was optimized in a previous study [6]. Briefly, the flow rate used was 400 *μ*L min^−1^ and the injection volume was 10 *μ*L. A binary gradient was used: acetic acid 0.1% (v/v) in water was used as mobile phase A, while acetic acid 0.1% (v/v) in methanol was used as mobile phase B. The analysis started with 95% mobile phase A. After 1 min, this percentage was linearly decreased down to 45% within 5 min. After that, solvent A was decreased quickly to 0% in 0.5 min and maintained for 1.5 min. The total run time was 20 min.

Mass analysis was performed on the Orbitrap mass spectrometer Exactive™ analyzer (Thermo Scientific, Bremen, Germany). The system was equipped with a heat electrospray ionization interface (HESI-II). The ion-source parameters were previously optimized as follows: spray voltage: 3.5 kV (positive mode) and 2.5 kV (negative mode); sheath gas flow rate: 55; auxiliary gas flow rate: 10; skimmer voltage: 23 V; heater temperature: 300°C; capillary temperature: 150°C; capillary voltage: 45 V and tube lens voltage: 120 V. For more details of the HRMS analysis see Roca* et al*. (2014) [6].

### 2.3. Compound Identification Criteria

The criteria for target compound identification were established following the SANTE/11813/2017 guideline [30]: (i) mass accuracy of the molecular ion< 5 ppm; (ii) mass accuracy of the fragment ion < 5 ppm; (iii) isotopic pattern similar to the theoretical isotopic pattern (the relative intensity of the A+1 and/or A+2 isotope peaks in the real sample shall correspond to the theoretical relative intensities). For confirmation we used the reference standard solutions of those compounds available in the market. In this case the confirmation criteria were included: (iv) retention time (t_R_) similar to that of the reference standard ± 0.1 min.

### 2.4. HRMS Orbitrap Parameters Optimization

#### 2.4.1. Resolving Power Optimization

To optimize the Resolving Power (R), the system operating in full-scan mode (50-800 m/z) was tested at the R of 10,000; 25,000; and 50,000 FWHM. 6 blank matrix urine aliquotes spiked with a mixed-standard solution of 24 target pesticide metabolites (see Table 1) (50 ng·mL^−1^) were analyzed.

The Resolving Power was evaluated measuring the peak area (signal intensity) and the mass accuracy (Δm) for the diagnostic and fragment ions of each metabolite. A scheme of the Resolving Power optimization study is detailed in Table SI.1. Resolving Power optimization data were acquired with ESI+/- in separated injections, using HCD fragmentation 20 eV and external mass calibration.

#### 2.4.2. Fragmentation Optimization

After the selection of the most suitable R, HCD and CID fragmentations were evaluated. Five spiked urine samples were injected with CID energies of 10, 20, 30, and 40 eV. We previously set the energy for HCD fragmentation to 20 eV. Once the CID energy was optimized, five different methods, in six spiked samples (50 ng·mL^−1^), were studied using or not HCD and CID fragmentations and using ESI+ and ESI- in the same or in different injections. The response was evaluated measuring the peak area of the fragment ions in (i) ESI^+^ with and without HCD (HCD= 20 eV); (ii) ESI^−^ with and without HCD (HCD= 20 eV); (iii) ESI^+^ with and without CID; (iv) ESI^−^ with and without CID; (v) ESI^+^ and ESI^−^ in the same injection with and without CID. Fragmentation optimization data were acquired using the previously optimized R and external mass calibration.

#### 2.4.3. Internal and External Mass Calibration Study

With respect to mass calibration, both external and internal mass calibrations were evaluated. External calibration was performed using the mixtures ProteoMass™ LTQ/FT-Hybrid ESI Cal Mix in Pos an Neg Mode (Supelco, Bellefonte, PA, USA). Internal mass calibration was achieved introducing caffeine (M+H^+^ m/z = 195.08765 Da) in the mobile phase as a lock mass for positive ionization (ESI^+^).

In total, five spiked aliquotes were analyzed using internal and external mass calibration separately. The analytical response was evaluated measuring peak areas and mass accuracies (Δm) for the diagnostic and fragment ions. All mass calibration study data were acquired using the previously optimized R and fragmentation settings.

Data were processed using the TraceFinder™ 3.1 (Thermo Scientific, Bremen, Germany) and Xcalibur™ 2.2 (Thermo Scientific, Bremen, Germany) software.

## 3. Results and Discussion

### 3.1. Resolving Power Optimization

In order to select the most appropriate R for the determination of pesticide metabolites in urine, the influence of this parameter on the signal and mass accuracy of the 24 compounds was investigated. Tables 2 and 3 show the signal intensity (peak area) and the mass accuracy (∆m), respectively, of the diagnostic and fragment ions.

Regarding diagnostic ions, some specific metabolites such as DEAMPY, IMPY, PNP, TCPy, MNP, DEP, DETP, or DIMET presented similar areas at the three R checked (Table 2). However, R = 25,000 FWHM provided the largest areas for DMDTP, MMP, OMET, cis DCCA, DBCA, ATZM, ALAM, METM, and 2,4,5-T. For the remaining 7 compounds a R= 25,000 FWHM presented areas close to that provided by the best R for each compound (10,000 FWHM or 50,000 FWHM).

For fragments, 17 out of 20 compounds presented higher intensities with R = 50,000 FWHM (Table 2). In some cases, as for METM, fragment ions areas are very low in comparison with the diagnostic ion response. It is relatively frequent that AIF produces fragment ions with low abundances, partly because the optimal fragmentation energy is not equal for all diagnostic ions. It could affect the analysis of real samples, because some diagnostic ions may not be confirmed because their fragment ions signal is below the noise. To sum up, R = 25,000 FWHM seems more suitable for diagnostic ions and 50,000 FWHM for fragment ions. However, taking into account the total ions (diagnostic plus fragments) and the variability of the response, a R = 25,000 FWHM was selected because it presented more number of ions with high area (see Figure SI.1). We have not found a clear explanation why some ions present higher responses when acquired at a R (e.g., 25,000 FWHM) while other ions present higher responses when acquired at another R (e.g., 50,000 FWHM).

In addition, the R of 25,000 FWHM presented more data points (scans) per peak than R = 50,000 FWHM because in an Orbitrap analyser the scan speed decrease when R increase (i.e., 2 Hz at R = 50,000 FWHM; 4 Hz at R = 25,000 FWHM). The number of data points is important for peak shape and quantification.

Table 3 shows the average and range of mass accuracy (∆m, ppm) of pesticide metabolites diagnostic and fragment ions obtained at the three R tested.  Table 4 summarizes the results showing the number of ions in each of the six ranges of mass accuracy considered. As can be observed (Table 4), good mass accuracies (<3 ppm) in most of the ions were obtained applying R of 25,000 and 50,000 FWHM.

Taking into account these results, a R = 25,000 FWHM was chosen because (i) it gave the highest signal (peak area) for more analytes; (ii) it presented good mass accuracy; and (iii) the scan time gave a suitable number of data points. An added advantage of using this intermediate R is that the instrument presents a sufficient speed to be able to use the detection of positive (ESI^+^) and negative (ESI^−^) ions in the same injection, increasing the speed of analysis and the throughput of the method. Martínez-Dominguez* et al*. (2016) also applied R = 25,000 FWHM for the analysis of organic contaminants in food using a UHPLC-Orbitrap detector [12]. QTOF systems also employed similar Resolving Powers (18,000-22,500 FWHM) for the analysis of pesticides, organophosphate flame retardants, and chemical agents in urine and drinking water [15, 16, 31]. However, in Orbitrap analyzer a higher Resolving Power (50,000 FWHM) was used for the analysis of pesticides and drugs in urine, food, and feed [6, 11, 27] and for the analysis of mycotoxins in food (70,000 FWHM) [14]. In contrast, a lower Resolving Power was employed in other studies ranging from 7,000 to 17,500 FWHM in biological and food samples [7, 12, 18]. Kaufmann* et al.* (2010) compared liquid chromatography selectivity in LC-MS/MS and LC-HRMS, applying different Resolving Powers (10,000-100,000 FWHM). They indicated that a Resolving Power of 50,000 FWHM was the most suitable for the analysis of veterinary drugs in food using LC-Orbitrap detector [22].

### 3.2. Fragmentation Optimization

**Figure 1:**
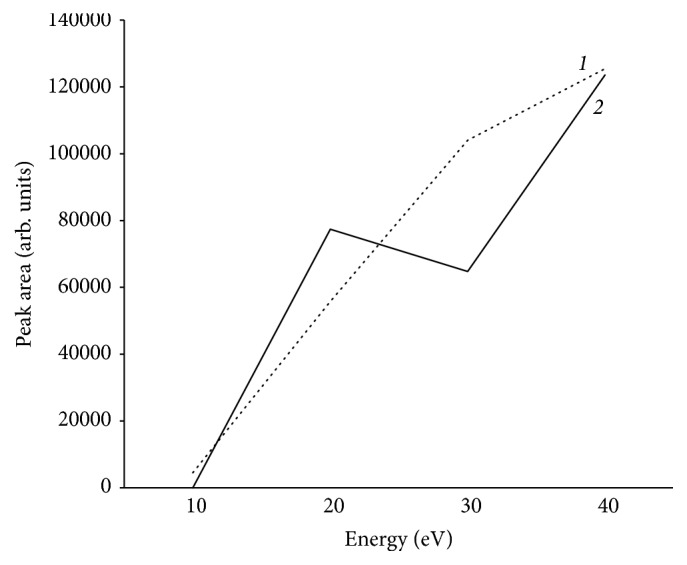
CID fragmentation of PBA (m/z = 169.06589). Variation of the fragment ion response (peak area) with the applied energy (eV). (1) Applying only ESI^+^; (2) using the polarity switching (ESI^+^, ESI^−^ in the same injection). Acquisition conditions: R= 25,000 FWHM and external mass calibration.

Regarding the optimization of the CID energy, the highest fragment areas were obtained using a fragmentation energy of 40 eV. As an example, Figure 1 shows the fragment ion areas obtained using CID at different energies for PBA.

**Figure 2:**
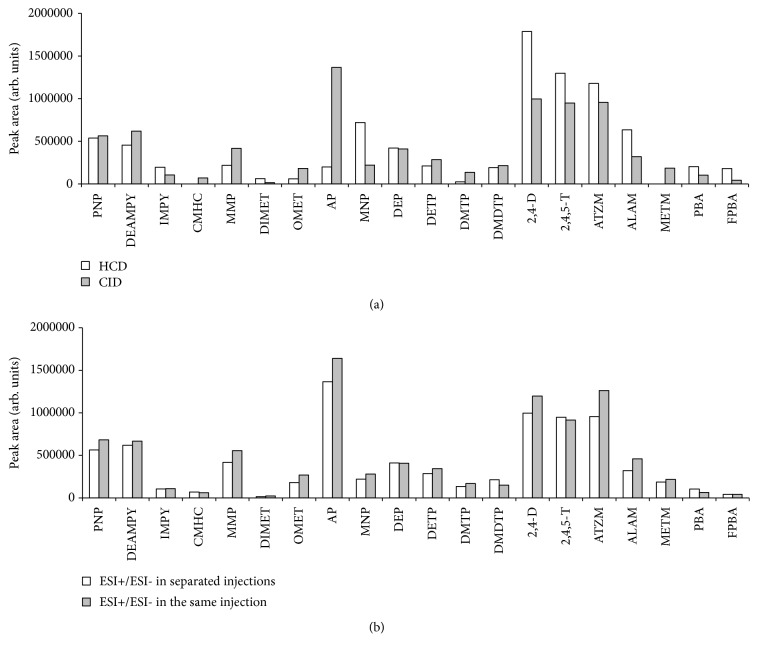
(a) Comparison between responses (peak area) of fragments obtained using CID (40 eV) and HCD (20 eV). (b) Comparison between CID fragmentation (40 eV) using the polarity switching function (ESI^+^ and ESI^−^) in the same injection and in two separate injections. Acquisition conditions: R= 25,000 FWHM and external mass calibration.

Comparing the results obtained using CID and HCD, Figure 2(a) shows similar areas for fragment ions obtained with CID and HCD modes. However, some compounds such as CMHC and METM were only fragmented applying CID. Consequently, CID energy (40 eV) was selected for all compounds. Similar results were obtained using ESI^+^ and ESI^–^ in the same injection than in two separate injections (see Figure 2(b)). Consequently, a method with ionization mode in ESI^+^ and ESI^–^ in the same injection and CID fragmentation was selected. Strano-Rossi* et al*. (2015) also selected CID energy (40 eV) in LC-Orbitrap for the determination of stimulants and drugs in food supplements [27]. Tak* et al*. (2014) employed higher CID energy (150 eV) in an LC-QTOF detector for the determination of chemical warfare agents in drinking water [31]. However, HCD is the most widely fragmentation mode used in HRMS Orbitrap detectors. HCD energies from 20 to 30 eV have been applied to fragment compounds such as pesticides, biotoxins, mycotoxins, veterinary drugs, and other toxins in biological, food, and feed samples [6, 11, 12, 18, 20]. Optimization of the mode of fragmentation in Orbitrap detectors is also carried out in other fields as proteomics. Jedrychowski* et al*. (2011) have evaluated CID/HCD fragmentation for murine phosphoproteomics [28].

### 3.3. Internal and External Mass Calibration Study

In general, the use of lock mass (internal calibration) improves mass accuracy. In addition, in the present method some fragments are below 138.06619 Da, which is the low mass in the ESI^+^ external calibration solution; consequently a continuous correction of the acquired masses (internal calibration) could avoid an excessive mass drift.

In order to check whether this general rule is applicable to this particular application, we studied the influence of the calibration mode on the mass accuracy of eight substances analyzed in ESI^+^ mode, using caffeine as internal standard.  Table 5 shows the mass accuracies (Δm, ppm) for the diagnostic and fragment ions obtained with internal and external mass calibration. As it can be observed, a better Δm was obtained when working with internal mass calibration. Hence, the use of caffeine as lock mass was selected.

In general, external mass calibration is used in the literature for HRMS Orbitrap detector and internal mass calibration for HRMS QTOF detectors. External mass calibration of Orbitrap in urine, plasma, food, and feed has been widely performed for pesticides, parabens, veterinary drugs, biotoxins, mycotoxins, and other substances [6, 8, 11, 12, 18, 20]. However, internal mass calibration has also been used, as in the method described by Strano-Rossi* et al*. (2015), for the analysis of various stimulants in food supplements. In this case the authors used diisooctyl phthalate ionic species, m/z 391.2843 Da, in order to compensate any possible mass axis drifts, obtaining ∆m < 1 ppm for all ionic species [27]. In contrast, QTOF detectors normally calibrate with negative internal mass calibration (leucine enkephalin) [15, 16, 19] or positive internal mass calibration (purine and hexakis-(1H,1H,3H-tetrafluoro-pentoxy)phosphazene) [31].

## 4. Conclusions

Mass spectrometry parameters such as Resolving Power, fragmentation mode, and type of mass calibration have been optimized for the analysis of 24 pesticide metabolites in urine. A Resolving Power of 25,000 FWHM, internal calibration, and CID fragmentation were selected as the best options to improve the signal intensity and the mass accuracy of diagnostic and fragment ions. This Resolving Power provides enough resolution to avoid isotopic interferences and allows the use of the polarity switching function (ESI^+^ and ESI^–^ in the same injection), hence reducing the analysis time.

The optimized HRMS parameters allow the determination of pesticide metabolites in urine samples; however a further validation of the method is required to determine the LOD and other performance parameters.

## 5. Study Limitation

We have not tested CID energies higher than 40 eV.

## Figures and Tables

**Table 1:** Pesticide metabolites: diagnostic and fragment ions used for analysis.

Class	Compound	Metabolite	Acronym	Elemental composition	Diagnostic ion	Exact mass m/z diagnostic ion (Da)	Fragment elemental composition	m/z fragment ion (Da)
Organophosphate insecticides	Chlorpyrifos, chlorpyrifos-methyl	3,5,6-Trichloro-2-pyridinol	TCPy	C_5_H_2_NOCl_3_	[M-H]^−^	195.91292	-	-
	Parathion, methyl parathion	p-nitrophenol	PNP	C_6_H_5_NO_3_	[M-H]^−^	138.01966	C_6_H_4_O_2_	108.02167
	Pirimiphos-methyl	2-Diethylamino-6-methyl-6-hydroxypyrimidine	DEAMPY	C_9_H_15_N_3_O	[M+H]^+^	182.12879	C_7_H_12_N_3_O	154.09748
	Diazinon	2-Isopropyl-4-methyl-6-hydroxypyrimidine	IMPY	C_8_H_12_N_2_O	[M+H]^+^	153.10224	C_4_H_6_NO	84.04439
	Coumaphos	3-Chloro-7-hydroxy-4-methylcoumarin	CMHC	C_10_H_7_ClO_3_	[M-H]^−^	209.00109	C_9_H_5_O_2_	145.02841
	Fenitrothion	3-Methyl-4-nitrophenol	MNP	C_7_H_7_NO_3_	[M-H]^−^	152.03531	C_7_H_6_O_2_	122.03733
	Dimethoate	Dimethoate	DIMET	C_5_H_12_NO_3_PS_2_	[M+H]^+^	230.00690	C_2_H_6_O_2_PS	124.98206
	Omethoate	Omethoate	OMET	C_5_H_12_NO_4_PS	[M+H]^+^	214.02974	C_4_H_8_O_4_PS	182.98754
	Acephate	Acephate	AP	C_4_H_10_NO_3_PS	[M+H]^+^	184.01917	C_2_H_8_O_3_PS	142.99262
	Methamidophos	Methamidophos	MMP	C_2_H_8_NO_2_PS	[M+H]^+^	142.00861	-	112.01577
	Chlorethoxyphos, chlorpyrifos coumaphos, diazinon, disulfoton, ethion, parathion, phorate, phosalone, sulfotep, terbufos, azinphos-methyl, dichlorvos, dicrotophos, dimethoate, fenitrothion, fenthion, malathion, methyl parathion, trichlorfon, chlorpyrifos-methyl, methidathion, mevinphos, oxydemeton-methyl, phosmet, pirimiphos-methyl, temephos, tetrachlorvinphos, isazofos-methyl, naled	Diethyl phosphate	DEP	C_4_H_11_O_4_P	[M-H]^−^	153.03221	C_2_H_6_O_4_P	125.00092
		Diethyl thiophosphate	DETP	C_4_H_11_O_3_PS	[M-H]^−^	169.00937	C_2_H_6_O_3_PS	140.97807
		Dimethyl thiophosphate	DMTP	C_2_H_7_O_3_PS	[M-H]^−^	140.97807	CH_3_O_3_PS	125.95460
		Dimethyl dithiophosphate	DMDTP	C_2_H_7_O_2_PS_2_	[M-H]^−^	156.95523	CH_3_O_2_PS_2_	141.93174

Phenoxy herbicides	2,4-Dichlorophenoxyacetic acid	2,4-Dichlorophenoxyacetic acid	2,4-D	C_8_H_6_O_3_Cl_2_	[M-H]^−^	218.96212	C_6_H_3_OCl_2_	160.95664
	2,4,5-Trichlorophenoxyacetic acid	2,4,5-Trichlorophenoxyacetic acid	2,4,5-T	C_8_H_5_O_3_Cl_3_	[M-H]^−^	252.92315	C_6_H_2_OCl_3_	194.91767

Chloroacetanilide herbicides	Atrazine	Atrazine mercapturate	ATZM	C_13_H_22_N_6_O_3_S	[M+H]^+^	343.15468	C_8_H_16_N_5_S	214.11209
	Alachlor	Alachlor mercapturate	ALAM	C_19_H_28_N_2_O_5_S	[M+H]^+^	397.17916	C_5_H_8_N O_3_	130.04987
	Metolachlor	Metolachlor mercapturate	METM	C_20_H_30_N_2_O_5_S	[M+H]^+^	411.19481	C_15_H_24_N O_2_S	282.15223

Pyrethroid insecticides	Commercial Pyrethroids	3-Phenoxybenzoic acid	PBA	C_13_H_10_O_3_	[M-H]^−^	213.05571	C_12_H_9_O	169.06589
	Cyfluthrin	4-Fluoro-3-phenoxybenzoic acid	FPBA	C_13_H_9_FO_3_	[M-H]^−^	231.04629	C_12_H_8_OF	187.05647
	Permethrin, cypermethrin, cyfluthrin	cis-(2,2-Dichlorovinyl)-2,2-dimethylcyclopropane-1-carboxylic acid	cis-DCCA	C_8_H_10_O_2_Cl_2_	[M-H]^−^	206.99850	-	-
		trans-(2,2-Dichlorovinyl)-2,2-dimethylcyclopropane-1-carboxylic acid	trans-DCCA	C_8_H_10_O_2_Cl_2_	[M-H]^−^	206.99850	-	-
	Deltamethrin	cis-(2,2-Dibromovinyl)-2,2-dimethylcyclopropane-1-carboxylic acid	DBCA	C_8_H_10_O_2_Br_2_	[M-H]^−^	294.89747	-	-

Internal Standards			PNP-D4	C_6_HD_4_NO_3_	[M-H]^−^	142.04477	-	-
			FPBA-13C6	^13^C_6_C_7_H_9_FO_3_	[M-H]^−^	237.06642	-	-
			DCCA-13C2	^13^C_2_C_6_H_9_DCl_2_O_2_	[M-H]^−^	210.01149	-	-
			ATZM-13C3	_13_C_3_C_10_H_22_N_6_O_3_S	[M+H]^+^	346.16475	-	-
			2,4-D-D3	C_8_H_3_Cl_2_O_3_D_3_	[M-H]^−^	221.98095	-	-
			MMP-D6	C_2_H_2_NO_2_PSD_6_	[M+H]^+^	148.04627	-	-
			DIMET-D6	C_5_H_6_NO_3_PS_2_D_6_	[M+H]^+^	236.04455	-	-
			DBP	C_8_H_19_O_4_P	[M-H]^−^	209.09481	-	-

**Table 2:** Average peak area and coefficient of variation (CV, %) obtained at 3 different Resolving Powers (R) for pesticide metabolites diagnostic and fragment ions (n = 6).

Metabolite	Peak area	Diagnostic ion			Fragment ion		
		R=10,000	R=25,000	R=50,000	R=10,000	R=25,000	R=50,000
CMHC	Average	908337	839333	570456	12927	26726	39492
	CV (%)	1.51	2.91	7.49	24.38	21.36	52.87
DEAMPY	Average	994268	880944	834834	63190	161187	175626
	CV (%)	17.15	28.51	10.49	22.31	16.20	40.94
IMPY	Average	1447089	1425080	1663104	Not found	47097	116409
	CV (%)	0.36	7.92	13.22	Not found	1.23	41.28
PNP	Average	6388647	6280026	6007343	3938342	3858484	4005830
	CV (%)	1.70	2.06	1.92	7.36	4.40	6.67
TCPy	Average	1090750	1148221	943686	-	-	-
	CV (%)	1.73	7.04	8.08	-	-	-
MNP	Average	3938892	3813784	3650660	503016	474896	634023
	CV (%)	3.10	3.85	4.18	11.40	13.27	19.46
DMTP	Average	76340	68339	88833	2644	6361	9973
	CV (%)	39.41	5.74	16.55	57.74	37.34	57.74
DMDTP	Average	111502	321392	247711	2679	16962	53675
	CV (%)	36.39	10.13	30.71	57.74	110.51	11.56
DEP	Average	1719737	1870997	1556112	196269	272546	272666
	CV (%)	4.09	4.33	5.83	21.00	12.49	6.12
DETP	Average	2224008	2154624	1819644	50934	141703	169339
	CV (%)	0.08	6.97	6.56	63.42	24.47	24.00
AP	Average	58729	74307	90733	157684	157729	206069
	CV (%)	8.34	33.62	17.63	7.21	4.59	13.43
MMP	Average	69818	178190	112470	19869	169131	69306
	CV (%)	7.38	2.89	4.58	25.93	3.05	7.43
OMET	Average	45484	226016	149173	13032	25505	32670
	CV (%)	69.04	23.35	17.68	35.04	22.09	32.95
DIMET	Average	380660	399855	329466	60934	83874	87121
	CV (%)	8.48	11.44	14.44	6.51	10.55	31.27
PBA	Average	648211	571829	363693	121191	138365	166373
	CV (%)	8.82	15.34	16.11	11.30	20.44	23.68
FPBA	Average	612579	519635	405204	73743	82755	112916
	CV (%)	13.94	23.07	11.99	16.68	13.95	31.57
cis DCCA	Average	171464	273263	194626	-	-	-
	CV (%)	28.60	16.39	13.55	-	-	-
trans DCCA	Average	453810	436816	314120	-	-	-
	CV (%)	6.12	6.49	13.04	-	-	-
DBCA	Average	6904	32503	32185	-	-	-
	CV (%)	31.56	40.91	16.74	-	-	-
ATZM	Average	273078	1179224	756085	1314830^a^	1421843^a^	1252716^a^
	CV (%)	71.21	3.96	2.01	3.57	4.46	9.05
ALAM	Average	67659	687712	511658	831200^a^	1255816^a^	1242225^a^
	CV (%)	12.30	15.92	7.77	7.02	2.35	5.40
METM	Average	268530	644807	446473	2762	5381	6657
	CV (%)	8.80	4.93	9.49	57.74	57.74	57.74
2,4-D	Average	1234998	1225331	907979	1130961	1117200	1224757
	CV (%)	6.27	4.39	5.67	3.85	8.45	3.68
2,4,5-T	Average	731224	786035	477627	677796	718415	777837
	CV (%)	10.79	10.86	10.65	7.95	12.15	13.05

Acquisition conditions: ESI+/ESI- in separated injections, HCD fragmentation 20 eV, and external mass calibration.

-: no fragment ions monitored.

^a^Irregular peak shape caused by isobaric interferences. The measured areas could be affected by the interferences.

**Table 3:** Average and range of mass accuracies (∆m) (ppm) obtained at 3 different Resolving Powers (R) for pesticide metabolites diagnostic and fragment ions (n=6).

Metabolite	Δm (ppm)	Diagnostic ion			Fragment ion		
		R=10,000	R=25,000	R=50,000	R=10,000	R=25,000	R=50,000
CMHC	Average	2.27	1.38	1.24	3.11	2.52	3.36
	Range	1.60 – 2.75	0.07 – 2.32	0.93 – 1.65	1.61 – 4.16	1.10 – 3.81	3.33 – 3.40
DEAMPY	Average	1.09	2.68	1.30	3.19	1.70	1.44
	Range	0.05 – 2.58	1.65 – 3.66	0.73 – 1.90	1.70 – 4.53	0.69 – 3.37	0.49 – 2.38
IMPY	Average	1.81	1.56	1.33	> 5	1.61	1.99
	Range	1.25 – 2.14	1.36 – 1.76	1.26 – 1.36	> 5	1.35 – 1.90	1.90 – 2.07
PNP	Average	2.69	1.94	1.63	3.64	2.18	1.96
	Range	2.31 – 3.08	0.77 – 2.87	1.20 – 1.86	2.92 – 4.99	1.09 – 3.13	1.48 – 2.28
TCPy	Average	2.23	2.01	1.83	-	-	-
	Range	0.28 – 3.01	0.42 – 3.12	1.05 – 3.38	-	-	-
MNP	Average	2.59	1.32	1.59	2.36	1.18	1.89
	Range	2.56 – 2.66	1.05 – 1.56	1.15 – 2.06	1.70 – 2.93	0.88 – 1.70	1.62 – 2.11
DMTP	Average	0.79	0.66	2.43	2.41	1.02	1.28
	Range	0.11 – 1.40	0.34 – 1.09	1.96 – 2.82	1.51 – 4.52	0.75 – 1.19	0.08 – 2.78
DMDTP	Average	2.47	1.49	2.14	1.96	2.67	1.31
	Range	1.26 – 3.21	0.87 – 2.52	1.65 – 2.43	0.25 – 4.03	1.67 – 4.26	0.49 – 2.10
DEP	Average	2.66	2.02	1.84	1.88	2.05	2.35
	Range	1.43 – 3.26	1.16 – 2.84	1.44 – 2.14	0.89 – 2.81	1.03 – 3.11	1.98 – 2.71
DETP	Average	3.46	2.02	1.96	2.26	3.13	2.10
	Range	3.28 – 4.99	1.27 – 2.63	1.30 – 2.29	0.61 – 4.22	2.30 – 4.22	1.31 – 3.04
AP	Average	1.66	1.41	1.39	1.91	2.26	1.79
	Range	0.42 – 4.80	0.60 – 2.10	0.48 – 2.57	0.06 – 4.00	1.30 – 3.16	1.13 – 2.11
MMP	Average	2.21	1.67	1.69	3.06	1.67	3.45
	Range	0.28 – 4.14	1.24 – 2.43	0.92 – 2.69	2.62 – 3.49	1.24 – 2.43	3.22 – 3.63
OMET	Average	0.86	1.92	2.09	1.55	3.36	1.90
	Range	0.03 – 1.39	1.11 – 2.61	1.86 – 2.38	0.47 – 2.39	2.17 – 4.98	1.19 – 2.99
DIMET	Average	2.79	2.55	2.13	2.38	2.34	2.67
	Range	1.18 – 4.62	2.18 – 2.92	1.14 – 3.10	0.38 – 4.06	1.10 – 3.90	2.20 – 3.11
PBA	Average	1.79	1.33	1.12	2.25	1.55	1.77
	Range	1.25 – 2.11	0.18 – 2.85	0.68 – 1.97	1.65 – 3.25	0.28 – 3.53	1.18 – 2.26
FPBA	Average	1.17	2.09	1.38	3.10	1.85	1.41
	Range	0.15 – 1.80	0.80 – 3.49	0.66 – 2.41	2.09 – 4.20	0.30 – 3.46	0.10 – 1.92
cisDCCA	Average	1.84	1.42	1.76	-	-	-
	Range	1.20 – 2.31	0.31 – 2.18	0.61 – 2.67	-	-	-
transDCCA	Average	2.37	1.82	1.41	-	-	-
	Range	1.05 – 3.47	0.23 – 3.04	0.31 – 2.45	-	-	-
DBCA	Average	2.92	2.07	1.78	-	-	-
	Range	1.80 – 4.10	1.08 – 2.74	0.36 – 3.16	-	-	-
ATZM	Average	3.61	2.29	2.98	1.18	1.84	1.85
	Range	2.29 – 4.97	1.43 – 2.87	2.17 – 4.12	0.83 – 1.63	1.42 – 2.32	1.56 – 2.27
ALAM	Average	3.41	2.57	3.29	2.30	2.14	1.96
	Range	1.24 – 4.76	1.69 – 2.85	2.85 – 4.76	1.38 – 3.25	1.61 – 3.38	1.61 – 2.43
METM	Average	2.37	2.68	2.77	> 5	1.52	2.13
	Range	0.90 – 4.38	1.70 – 3.87	1.19 – 4.31	1.09 – >5	0.42 – 2.35	1.82 – 3.13
2,4-D	Average	2.65	1.92	1.72	3.21	2.19	1.89
	Range	1.98 – 3.62	0.97 – 2.56	0.74 – 2.77	2.82 – 3.87	1.08 – 3.00	1.76 – 2.14
2,4,5-T	Average	1.65	1.30	2.28	2.89	2.15	1.71
	Range	0.46 – 2.26	0.22 – 1.85	1.30 – 4.14	2.16 – 3.65	1.42 – 2.78	1.29 – 2.23

Acquisition conditions: ESI+/ESI- in separated injections, HCD fragmentation 20 eV, and external mass calibration.

-: no fragment ions monitored.

**Table 4:** Number of ions (diagnostic and fragment ions) into the six mass accuracy ranges considered at 10,000, 25,000, and 50,000 FWHM.

Resolving Power	∆m (ppm)					
	≤ 1	]1 – 2]	]2 – 3]	]3 – 4]	]4 – 5]	> 5
10,000	2	12	19	9	0	2
25,000	1	22	19	2	0	0
50,000	0	30	11	3	0	0

Acquisition conditions: ESI+/ESI- in separated injections, HCD fragmentation 20 eV, and external mass calibration.

**Table 5:** Average mass accuracies (∆m) (ppm) and standard deviations (Std dev) using internal and external calibration for diagnostic and fragment ions in ESI positive mode (n=5).

Metabolite	Diagnostic ion				Fragment ion			
	Internal calibration		External calibration		Internal calibration		External calibration	
	∆m (ppm)	Std dev	∆m (ppm)	Std dev	∆m (ppm)	Std dev	∆m (ppm)	Std dev
DEAMPY	0.21	0.13	0.47	0.26	0.17	0.08	1.15	0.19
IMPY	0.08	0.06	0.62	0.26	3.55	0.08	2.64	0.23
DIMET	0.27	0.07	0.76	0.29	0.44	0.05	0.50	0.12
OMET	0.14	0.10	0.53	0.39	0.12	0.11	0.90	0.33
AP	0.12	0.10	0.41	0.21	0.15	0.08	0.99	0.27
ATZM	0.49	0.15	0.99	0.41	0.19	0.04	0.91	0.36
ALAM	0.24	0.27	1.14	0.55	0.17	0.14	0.59	0.17
METM	1.52	1.38	2.23	1.07	0.77	0.45	1.96	0.39

Acquisition conditions: ESI+/ESI- in the same injection, R = 25,000 FWHM, and CID fragmentation 40 eV.

## Data Availability

The data used to support the findings of this study are available from the corresponding author upon request.

## Supplementary Materials

Supplementary Materials Table SI.1: scheme of the Resolving Power optimization study. Figure SI.1: number of ions with high ([100-80]%) and low (<80%) peak areas at R=10,000, R=25,000, and R=50,000 (FWHM), for total, diagnostic, and fragment ions.
