# Supplementary material for: Optimization of Resolving Power, Fragmentation, and Mass Calibration in an Orbitrap Spectrometer for Analysis of 24 Pesticide Metabolites in Urine
Source: Int J Anal Chem. 2019 Apr 17;2019:1917369. doi: 10.1155/2019/1917369 (PMC6500652; doi:10.1155/2019/1917369)
Supplement: Supplementary Materials — Table SI.1: scheme of the Resolving Power optimization study. Figure SI.1: number of ions with high ([100-80]%) and low (<80%) peak areas at R=10,000, R=25,000, and R=50,000 (FWHM), for total, diagnostic, and fragment ions. [file 1917369.f1.pdf]

Supplementary material

Optimization of Resolving Power, fragmentation and mass calibration in an Orbitrap Spectrometer for analysis of 24 pesticide metabolites in urine

Authors: Pablo Dualde [a, b], Clara Coscollà [a, c], Agustin Pastor [b] and Vicent Yusà* [a, b, c]

[a] Foundation for the Promotion of Health and Biomedical Research in the Valencian Region, FISABIO-Public Health, 21, Avenida Catalunya, 46020, Valencia, Spain;
[b] Analytical Chemistry Department, University of Valencia, Edifici Jeroni Muñoz, Dr. Moliner 50, 46100 Burjassot, Spain;
[c] Public Health Laboratory of Valencia, 21, Avenida Catalunya, 46020 Valencia, Spain.

Table of Contents

Type	Captions	Page
Table SI.1	Scheme of the Resolving power optimization study	1
Figure SI.1	Number of ions with high ([100-80]%) and low (<80%) peak areas at R=10,000, R=25,000 and R=50,000 (FWHM), for total, diagnostic and fragment ions.	2

Table SI.1. Scheme of the Resolving power optimization study

**Ionization mode**	**ESI NEG**			**ESI POS**		
**Resolving power (FWHM)**	**10,000**	**25,000**	**50,000**	**10,000**	**25,000**	**50,000**
**Nº aliquotes (spiked urine) injected[a]**	6	6	6	6	6	6
**Nº acquisitions non-applying fragmentation[a] (diagnostic ions)**	6	6	6	6	6	6
**Nº acquisitions applying fragmentation (HCD 20 eV)[a] (fragment ions)**	6	6	6	6	6	6

[a]Each injection allowed both “applying fragmentation” and “non-applying fragmentation” acquisitions quasi-simultaneously

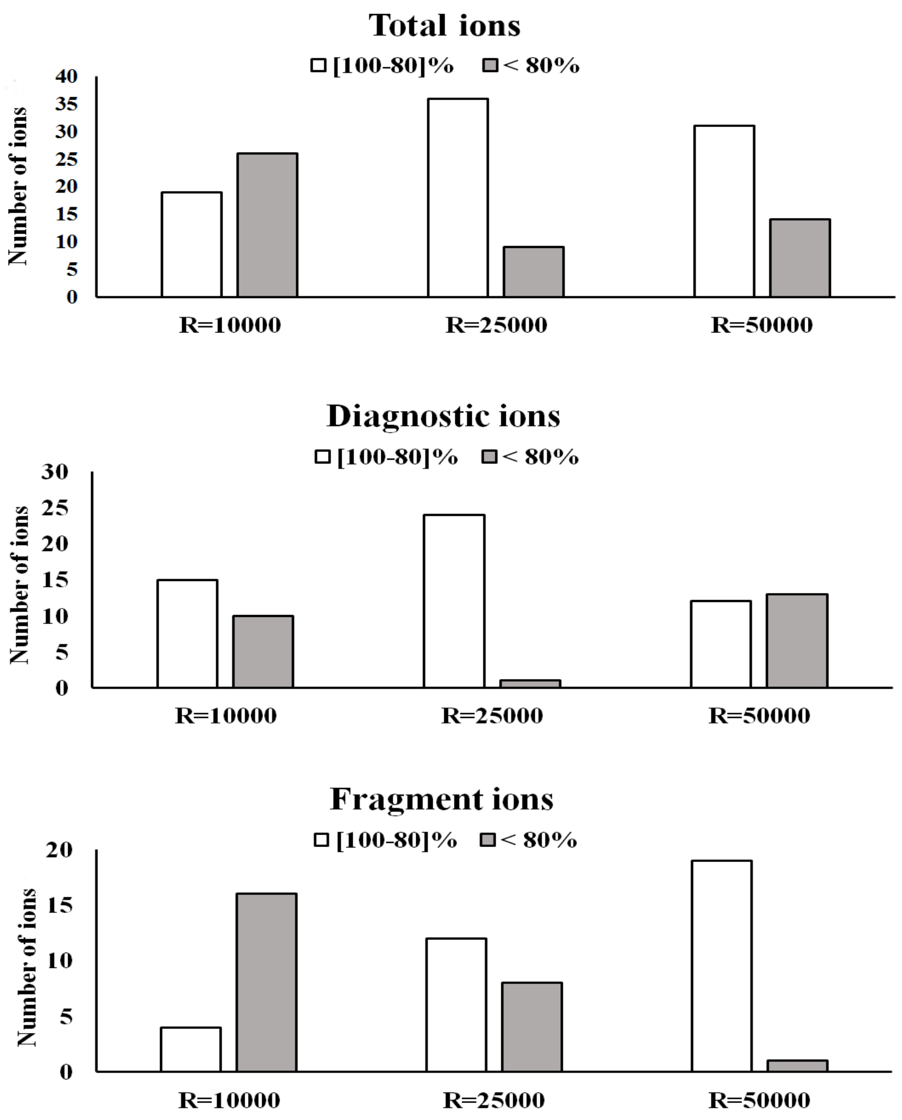

Figure SI.1. Number of ions with high ([100-80]%) and low (<80%) peak areas at R=10,000, R=25,000 and R=50,000 (FWHM), for total, diagnostic and fragment ions. The percentage (%) is calculated dividing the area of an ion by the highest area obtained for that ion and multiplying by 100.
